# An Improved Effective Cost Review Process for Value Engineering

**DOI:** 10.1155/2014/682051

**Published:** 2014-11-03

**Authors:** D. S. Joo, J. I. Park

**Affiliations:** Department of Industrial Engineering, Ajou University, San 5, Woncheon-dong, Yeongtong-gu, Suwon 443-749, Republic of Korea

## Abstract

Second-look value engineering (VE) is an approach that aims to lower the costs of products for which target costs are not being met during the production stage. Participants in second-look VE typically come up with a variety of ideas for cost cutting, but the outcomes often depend on their levels of experience, and not many good alternatives are available during the production stage. Nonetheless, good ideas have been consistently generated by VE experts. This paper investigates past second-look VE cases and the thinking processes of VE experts and proposes a cost review process as a systematic means of investigating cost-cutting ideas. This cost review process includes the use of an idea checklist and a specification review process. In addition to presenting the process, this paper reports on its feasibility, based on its introduction into a VE training course as part of a pilot study. The results indicate that the cost review process is effective in generating ideas for later analysis.

## 1. Introduction

Value engineering (VE) is a value-enhancing technique developed by Miles, of which cost cutting is a part [1]. It is a proven method for improving product and process performance by changing materials, designs, and systems and aims to maximize function while minimizing cost [2]. VE has been embraced by a global spectrum of businesses and industries, particularly in areas such as defense, transportation, construction, and healthcare [3].

VE has evolved to comprise three phases, which are sometimes collectively referred to as product development VE: (1) zero-look VE, (2) first-look VE, and (3) second-look VE [4]. Zero-look VE is the application of VE principles at the concept proposal stage. One of its objectives is to introduce new forms of functionality that did not previously exist. Once an overall concept has been established during zero-look VE, first-look VE focuses on the major elements of product design. The objective of second-look VE is to lower the costs of products at the production stage when target costs are not currently being met. During this stage, when products are already in production, not many good alternatives are available, and so companies are compelled to use second-look VE to lower costs.

Brainstorming is a commonly used method of generating ideas and has been adopted in VE in order to develop a wide variety of ideas in a constraint-free atmosphere. However, the ideas resulting from brainstorming in VE tend to be biased and limited, because participants rely on their experience in their areas of engineering focus [5]. In other words, experienced individuals often fail to explore other feasible, possibly better ideas in different engineering areas [6]. At the same time, it is difficult for entry-level participants to come up with many ideas without training, since they may have difficulty imagining and investigating ideas within their areas of expertise.

Asking questions that stimulate curiosity and creativity is a helpful approach to idea generation and makes it possible to be creative in a thorough and even orderly way. A written list of mentally stimulating questions can remind individuals of approaches and possibilities that they may otherwise not have had in mind [7]. A notable example of this can be seen in engineers' use of the “eliminate, combine, rearrange, and simplify” (ECRS) method to discover potential changes that they can make to an existing product, so as to create a better one. These proposed changes may either be in the form of direct suggestions or starting points for lateral thinking. However, ECRS is so general that the process often ends up leading to interesting but impractical and uneconomical ideas. In contrast, second-look VE professionals tend to come up with small and simple yet practical cost-cutting ideas that lower the costs of products during the production stage. ECRS alone is thus not particularly well suited to coming up with second-look ideas.

In general, it is well known that the best cost-cutting ideas for second-look VE are associated with eliminating unnecessary or unused portions of functions or overspecifications [8]. VE professionals have typically been trained and disciplined over several years, with the goal of boosting their ability to generate such ideas. They learn how to come up with more ideas, how to analyze methods for developing ideas, and how to recognize good ideas, tips, and techniques. The thinking processes performed by professionals during second-look VE involve recognizing specification differences [9]. This enables VE professionals to think of new directions and generate cost-cutting ideas that can be accepted for further analysis. If such thinking processes can be formulated, it can be very useful for engineers who have difficulty generating cost-cutting ideas.

This paper presents a cost review process that was developed as a supportive method for investigating cost-cutting ideas in second-look VE. For it, we investigated second-look VE cases for 20 home appliances, and created an idea checklist that was specific to those product models. In addition, we developed a specification review process to explore cost-cutting ideas by checking whether specifications are appropriate or can be improved. The rest of this paper is organized as follows. In Section 2, idea generation methods that are frequently used in VE are reviewed, with regard to their problems.  Section 3 explains how the idea checklist and specification review process for this study was developed and applied.  Section 4 presents the application of the proposed idea generation method to cases, while Section 5 evaluates the effectiveness of the method in a VE training course. The benefits and limitations of the proposed method are discussed in Section 6. Finally, Section 7 concludes with a summary and description of potential future work based on this study.

## 2. Idea Generation Methods

Much existing research on idea generation methods has focused on the goal of increasing the number of ideas produced, because it has been established that a direct relationship exists between the number of initial ideas and the quality of a final idea [10]. The main focus of such studies has been on brainstorming techniques for group-thinking sessions that increase the quality and quantity of proposed ideas for addressing a specific problem [11]. It has been claimed that group brainstorming is more effective than individual brainstorming, because mixing more different personalities into the creative flow results in a broader outlook in ideas, and the use of a facilitator to lead the session makes the process run more smoothly and removes potential bias [12]. As such, group brainstorming is the most common creativity technique used in the alternative phase of VE. Several books on VE, such as those of Fowler [13] and Dell'Isola [14], have presented brainstorming as the ideal creative technique for use in VE.

However, some VE researchers and professionals have questioned the usefulness of group brainstorming, claiming that it blocks the creative thinking efforts of a group [15]. VE participants are likely to experience the greatest difficulty during the process of idea generation. One problem is that participants tend to get stuck inside their own heads, in terms of their thinking and ideas, and do not exchange enough ideas through idea generation methods, due to perceptual blocks, emotional blocks, habitual blocks, professional blocks, or cultural blocks, which can hinder creative ability [16–18].

Individuals engaging in creative thinking should ideally engage naturally in a creative process that is as free of structure, rules, and processes as possible, but it is unlikely that a group will spontaneously do so in reality [19–21]. This is why applying a proven, systematic structure to brainstorming sessions significantly increases team productivity [22–24]. Al-Ghamdi [25] has investigated the necessary steps for effective implementation of brainstorming, and proposed a five-step procedure that helps to enhance the effectiveness of brainstorming implementation in VE studies. This study suggests that creative professionals prefer to use set methods to produce more creative results.

Asking questions to stimulate curiosity and creativity has proven to be a helpful method for idea generation, which shows that it is sometimes possible to be creative in a thorough and even orderly way. A written list of mentally stimulating questions can remind individuals of approaches and possibilities that they otherwise would not have considered. In such contexts, a checklist can play a key role in generating ideas [26]. Checklists can include ideas collected through previous experiences, so as to trigger team members' thinking about solutions to a problem under study. SCAMPER (substitute, combine, adapt, modify, put to other uses, eliminate, and rearrange) is an acronym for useful approaches that can be applied as stimuli to make one think differently about a problem area [27]. The SCAMPER method can be used to ask questions about existing products, using each of the approaches involved. Such questions are effective in helping individuals come up with creative ideas for developing new products or improving current ones.

Checklists may range from general to very specialized. General checklists are useful for almost any VE study. They contain suggestions for rearranging, substituting, and combining existing ideas in order to develop new ones. However, specialized checklists are only useful for addressing specific problems. For example, a series of questions can be created that stimulates new ways of thinking about new processes or devices [28]. In such cases, however, ordinary engineers might limit themselves to considering the listed questions, without giving much thought to other possible alternatives.

Brainstorming is often more effective if it is combined with other idea-generation techniques. These include the Delphi method, the Gordon technique, the morphological analysis technique, and TRIZ contradiction analysis. Of these, TRIZ provides the most structured and systematic approach and can be best used to augment or replace traditional brainstorming as it is currently applied in VE [29]. This makes the integration of VE and TRIZ beneficial, as it facilitates the transition from functional diagramming to implementable solution concepts [30, 31]. The value in using these methods is in how they include additional thoughts that inspire participants and allow their minds to wander or make intuitive leaps. However, these methods cannot ensure that the ideas that are sought out will be found.

## 3. Developing a Specification Review Method

This study presents the development of a cost review process that helps to investigate cost-cutting ideas for second-look VE. Its main focus is the development of an idea checklist and specification review process. The checklist is created by investigating previous second-look VE cases and classifying them according to idea stimuli, while the specification review process is developed based on the thinking processes engaged in by VE professionals.

### 3.1. Developing the Idea Checklist

**Figure 1:**
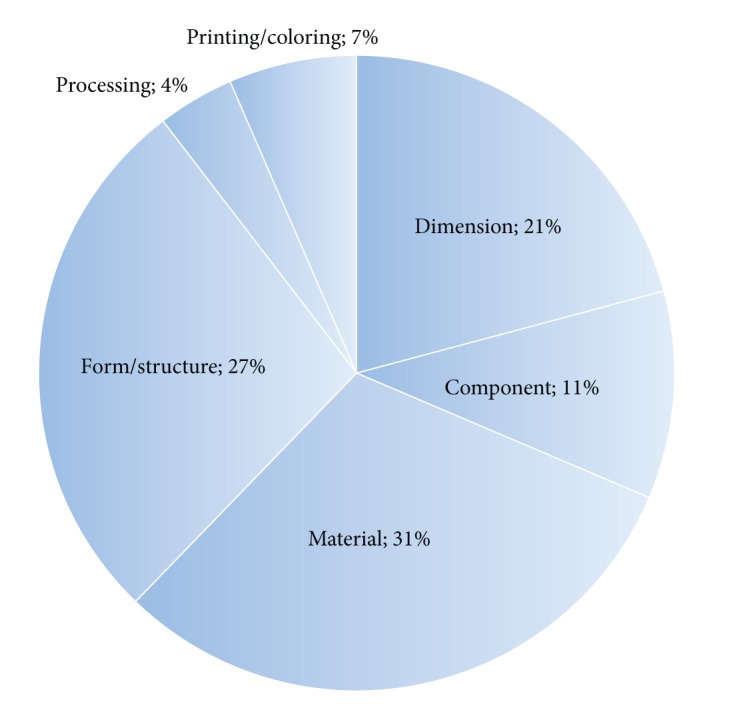
Six idea categories of second-look VE cases for home appliances.

For this study, we investigated the second-look VE cases of 20 home appliances, including TVs, refrigerators, washing machines, air conditioners, and peripheral computer devices. We organized 691 actual cases into eight idea categories: (1) material (213 cases), (2) form/structure (189 cases), (3) dimension (166 cases), (4) component (73 cases), (5) printing/coloring (45 cases), and (6) processing (27 cases). We created a checklist based on this classification by grouping similar cases and giving them appropriate names. As can be seen in Figure 1, the categories of material, form/structure, dimension, and component account for roughly 90% of the cases, while the other categories account for only roughly 10% of cases. Therefore, efforts to generate ideas through second-look VE should concentrate on these four categories, which describe the most frequently encountered instances.

#### 3.1.1. Material

*(i) Cost-Cutting Ideas*. These are the most frequently encountered ideas among VE cases. Their main concept is to meet prevailing functional requirements with less expensive alternative materials.  Table 1 presents material-related idea stimulus questions, which involve replacing the currently used material with one that is less costly. Since these ideas comprise the largest portion of cost-cutting ideas, they should be considered a priority in the exploration of cost-cutting ideas. To use a lower-grade material for its own sake is a bad idea. Therefore, engineers need to have considerable knowledge about material properties. There are two types of material-related ideas. The same material of a lower grade replacement is one that (a) uses the same material, (b) is of a lower grade, and (c) meets functional specifications. A different material at a lower cost replacement is one that (a) uses a different material, (b) costs less, and (c) meets functional specifications.

*(ii) Expression*. The cost-cutting ideas in this category take the form *〈*Category, Subcategory, Object (to object)*〉*, where (to object) concerns a potential anticipated object for a cost-cutting idea. A representative case of the same material of lower-grade replacement is the replacement of SUS 303 of a shaft with HIPS HB. This case can be represented as *〈*Material, Same material of a lower grade replacement, Shaft_SUS 303 (to SUS204CU)*〉*. A representative case of a different material at a lower cost replacement is the replacement of ABS plastic of a cover with HIPS. This case can be represented as *〈*Material, Different material at a lower cost replacement, Cover_ABS plastic (to HIPS)*〉*. If there is no potential object to be anticipated, this case can be represented as *〈*Material, Different material at a lower grade replacement, Cover_ABS plastic ( )*〉*. More examples can be found in Table 1.

#### 3.1.2. Form/Structure

*(i) Cost-Cutting Ideas*. The form/structure category accounts for the second-largest share of second-look VE cases. The main idea of form and structure cost-cutting ideas is to eliminate, combine, rearrange, and simplify some aspect of the form or structure of an existing product while meeting functional requirements.  Table 2 shows idea stimulus questions and cases that can be classified as form/structure cost-cutting ideas. These ideas are meant to reduce material or processing costs by altering a component's form or structure.

*(ii) Expression*. A representative case of simplification is that of simplifying the comb pattern ribs of a friction holder rear by replacing it with simple pattern ribs. This case can be represented as *〈*Form/Structure, Simplification, Fiction holder rear_comb pattern ribs (to simple pattern ribs)*〉*. If there is no potential object to be anticipated, this case can be represented as *〈*Form/Structure, Simplification, Friction holder rear_comb pattern ribs ( )*〉*. More examples are found in Table 2, and some cases are illustrated with pictures as shown in Table 3.

#### 3.1.3. Dimension

*(i) Cost-Cutting Ideas*. These ideas are meant to reduce the size or weight of an existing product in order to cut material costs. Dimension-related features include thickness, length, width, height, and diameter. Reducing the thickness of a component is one of the most frequently used ideas for cutting costs related to dimension. These idea stimulus questions are shown in Table 4.

*(ii) Expression*. The cost-cutting ideas in this category take the following form: *〈*Category, Subcategory, Object_(target value)*〉*, where a target value is a potential one anticipated. A representative case of cost cutting with regard to thickness involves reducing an inlay panel's 0.25 t by 0.17 t. This case can be represented as *〈*Dimension, Thickness reduction, Inlay panel_0.25 t (to 0.17 t)*〉*. More examples can be found in Table 4.

#### 3.1.4. Component

*(i) Cost-Cutting Ideas*. The major ideas in this category are meant to cut costs by finding lower-grade or otherwise lower-cost components as substitutes for existing components. The minor ideas involve reducing the number of components (e.g., PCB test pins, connector pins, holes) or their capacities (e.g., volts, memory size). These idea stimulus questions are shown in Table 5. To find proper substitute components, engineers need sufficient knowledge to find those that are cheap but suitable and available, while meeting prevailing functional requirements.

*(ii) Expression*. A representative case of the same component of a lower grade component replacement is the replacement of the 2012 cable of an SMD chip resister with a 1608 cable. This case can be represented as *〈*Component, Same component of a lower cost replacement, SMD Chip Resister_2012 cable (to 1608 cable)*〉*. More examples can be found in Table 5.

#### 3.1.5. Printing/Coloring

*(i) Cost-Cutting Ideas*. The ideas in this category involve the application of low-cost printing and coloring methods. The basic questions in this category concern the possibility of applying low-cost printing methods or omitting coloring processes by using natural or the same (existing) color. Table 5 shows idea stimulus questions related to labeling and painting.

*(ii) Expression*. A representative case of lower-cost printing replacement involves replacing the labeling of an internal case with carved sealing. This case can be represented as *〈*Printing/coloring, Lower cost printing replacement, Internal case_labeling (to carved sealing)*〉*. More examples can be found in Table 6.

#### 3.1.6. Processing

*(i) Cost-Cutting Ideas*. These ideas are meant to reduce processing time by changing a joint type, eliminating an assembly step, or reducing a processing step.  Table 7 shows idea stimulus questions related to processing.

*(ii) Expression*. A representative case of lower-cost processing is the replacement of the screws of a main PCB and a boy frame with tapes. This case can be represented as *〈*Processing, Joint type replacement, Main PCB and body frame joint_screws (to tapes)*〉*. More examples can be found in Table 7.

Table 8 summarizes an idea checklist that was created by classifying second-look VE cases according to idea stimuli. Engineers can work through the checklist in brainstorming sessions in which they examine a product for which target costs are not currently being met. They can answer the questions in the checklist one by one while examining the product. This idea checklist can thus be a supportive tool for reminding inexperienced engineers of various idea perspectives to consider. However, the list may not by itself be able to help engineers think of more practical ideas. A systematic process is needed to make ideas more practical.

### 3.2. Developing the Specification Review Process

For this study, we interviewed VE professionals who had 15–20 years of experience about how to find direction in cost-cutting ideas during second-look VE. We found that design specifications are used to generate, test, and evaluate design ideas, because they are essential qualitative and quantitative characteristics for setting criteria to be satisfied in designing a component, product, or system (such as performance requirements, dimensions, weight, or reliability). The review processes performed by professionals involve investigating current specifications based on the assumption that design specifications may have been decided without adequate review. This kind of thinking process can be very useful for engineers who have difficulty generating cost-cutting ideas.

Table 9 presents the kind of specification review process that is typically carried out by VE professionals. In the first step, the specifications of components are identified and recorded with drawings or approved calculation sheets. If there are no existing specifications, the specifications need to be established through the proper methods. In the second step, all relevant regulations are explored, such as laws, legal standards, environmental regulations, safety standards, or service guidelines. By looking into issues of who created the specifications, how, and why, more information can be obtained. If reasons and methods for creating specifications can be determined, they can help professionals understand historical approaches. If they cannot be determined, it can be concluded that specifications have been created without an adequate review process. In the third step, cost-cutting ideas are generated based on determining an idea form: *〈*Category, Subcategory, object (to object)*〉*. This aims to find any potential problems that could arise from object to object and to determine how to overcome them. Sometimes, these ideas can be obtained simply by benchmarking.

Going through steps 1, 2, and 3 makes it possible for ideas to be explored and expanded on. This review process, together with the idea checklist, allows brainstorming to be conducted more effectively.

In summary, the cost review process consists of two steps: identifying the relevant subcategories within the idea checklist through a brainstorming session and then conducting a specification review for the subcategories identified as relevant. During the brainstorming session, VE participants investigate a product for which cost targets are not currently being met by going through the idea checklist and identifying potential subcategories for cost cutting. In the subsequent specification review process, they identify the current specifications, explore requirements and the issues of “who, why, and how,” and think of cost-cutting ideas for the subcategories.

## 4. Implementation

In the next step in this study, the idea checklist and design specification review described in Section 3 were implemented in a training course for the associate value specialist (AVS) program in a company. The AVS program is a certificate program for engineers who decide to become professional value engineers [32]. The program is based on a process known as a multistage job plan, which provides structure for value studies. The plan consists of six sequential steps: (1) the information phase, (2) the function analysis phase, (3) the alternative phase, (4) the evaluation phase, (5) the development phase, and (6) the presentation and implementation phase. The goal of the plan is to generate as many practical ideas as possible for later analysis. This shows that idea generation can be seen as the most important part of VE. This paper only describes the phase associated with the proposed method in the training course.  Table 10 shows the appearance and component configuration of a handset used in this training. A hierarchical decomposition of the product or what is typically known as the bill of materials (BOM) is also shown in Table 10. Two cases were selected to demonstrate how the supportive method is implemented.

### 4.1. Implementation Case I

The proposed method was applied to handset cases ① and ② in Table 10, in order to demonstrate how it works. The function of the handset cases was to protect the internal parts of the handset, and their target costs were not being met. Few good alternatives were available, and so an idea checklist was employed to generate cost-cutting ideas. By checking the idea stimuli one at a time in a brainstorming session, four subcategories were developed by the engineers in the training program, as shown in Table 11.

In the second round, a specification review was conducted to explore the four subcategories based on specification data. The current design specification of the handset cases with respect to material is standard ABS (acrylonitrile butadiene styrene) V0. With regard to thermal characteristics, the regulations imposed on handset uppers are the UL (Underwriters' Laboratories) and TUV (Technischer Überwachungs Verein) standards. The history of the design was explored, and it was found that the existing design specification involved using heat-resistant resin, as imposed by UL/TUV standards, primarily to impede fires in the phone line. The specification review resulted in examining the following idea form: *〈*Material, Same material of a lower grader replacement, Handset cases_ABS (to ABS HB)*〉* by questioning what problems you expect and how you overcome them, because ABS HB (hard board) has the same functional specifications as ABS V0, and is also heat-resistant.

The next subcategory concerned the thickness of the handset cases. The written specifications for the cases describe a thickness of 2.5 mm, as in the schematic drawing. However, the measured thickness of the handset was 2.8 mm, which was over specifications. The safety regulations imposed on handset cases dictate that they must not break under 5Ns impact. Furthermore, a review of the history of the handset design revealed that the existing design specifications had been set up without adequate specification review. The specification review resulted in examining the following idea form: *〈*Dimension, Thickness reduction, Headset case_0.28 t (to 0.25 t)*〉*. It should have investigated the thickness needed to withstand the impact of 5 Ns. The application of an appropriate specification review process to the handset cases is summarized in Table 12.

### 4.2. Implementation Case II

Table 13 shows an example of idea generation for the PBA SUB-etc. MIC (⑥) and CFH (⑨) in Table 10, for a function in which an electrical signal is passed to the modular jack. Four subcategories were chosen from the idea checklist for further investigation.

A specification review was conducted to further explore the four subcategories in a systematic manner, based on specification data. The form/structure subcategory concerned the integration of the PBA SUB-etc. and CFH. In the existing structure, the CFH and the PCB-handset MIC were detachable at the modular jack, with the modular jack soldered onto the PCB-handset MIC in PBA SUB-etc. The safety regulations imposed on the CFH were unknown. The actual structure of the CFH and the PCB-handset MIC can be split. An examination of the history of the design revealed that the existing structure was set up by an engineer due to problems related to repairs after sales. Therefore, the relevant after-sales claim rates need to be investigated to decide whether the CFH and the PCB-handset MIC should be separated. The idea generated from this specification review was to examine the feasibility of soldering the CFH directly to the PCB-handset MIC without the modular jack, if rates of after-sales repairs were low or nonexistent.

The second subcategory is related to the length of the CFH. The existing length of the CFH was 300 ± 20 cm. The actual measured length was 305 cm. The safety regulations imposed on the CFH were unknown. A review of the history of the design showed that the existing specifications had been set without adequate specification review. The actual length is within the given range, meaning that the current length has been set appropriately with respect to the current specifications. The idea generated from this specification review was to examine the length of other commercial models in order to benchmark them for comparison with the current design specification. The application of the specification review process to the PBA SUB-etc. and CFH is summarized in Table 14.

## 5. Evaluation

A preliminary evaluation of the proposed method for brainstorming was conducted by implementing the method in a training course, in order to determine whether it could be used as a supplementary method to help engineers generate ideas. We assembled ten brainstorming groups. Each group was made up of 4-5 persons. We introduced the purpose of the test and provided the method, along with the product. The experiment was conducted just after brainstorming, without applying the method. The results of the preliminary test are shown in Table 15.

The total numbers of ideas generated by brainstorming without and with the supportive method were 75 and 219, respectively. Through the use of the supportive method, the average number of ideas improved from 7.5 to 21.9, which is an increase of 293%. These results of the preliminary test result suggested that the use of the supportive method for brainstorming resulted in training outcomes that were significantly better than those achieved through brainstorming alone.

A most significant observation was made during the test. When running through the checklist, the leader of one group asked, “Taking this idea checklist into account, how might I check this specification? What effect will this idea have on the problem?” as well as other similar questions. The other members of the group answered the questions. We observed that the checklist stimulated the participants, who had trouble remaining alert during the brainstorming session in which the supportive method was not used.

## 6. Discussion

Our evaluation of the training effectiveness of the method showed that participants felt that it made positive contributions to their brainstorming behaviors. This suggests that the method reinforces brainstorming by providing a more efficient approach to generating ideas. However, our interviews with several VE experts revealed a somewhat different reality.

For our study, we also examined people's attitudes toward this method. In a postexperimental survey, eight of 10 groups said that the method was either helpful (six groups) or sometimes helpful (two groups). Some participants informally praised the method, and one volunteered that the method was helpful for him because the idea checklist and review process smoothly guided idea generation. Another got quite excited about the effectiveness of the method and stated that he planned to introduce it to his team members, who work on home appliance development. However, some participants criticized the method, arguing that the idea stimuli such as dimension, amount, and size are well-known approaches in VE, and thus the method is only effective for entry-level engineers. For senior engineers who have experience in their fields, the method may even limit idea generation. Hence it is necessary that engineers expand on checklists with as many feasible alternatives as possible.

In general, a specification review is an important part of second-look VE. It provides essential technical details on the materials or processes to be used in developing a product that conforms to customer requirements. Product nonconformance is often associated with inadequate specification review. Engineers often set design specifications without adequate specification review due to a lack of time and information. Problems of this type can be avoided with a well-planned and managed specification review procedure. Therefore, specification reviews are a necessary process for further exploring ideas that need to be visualized by engineers.

## 7. Conclusion

This study presents a supportive method for supplementing brainstorming activities that do not play a role as starting points for second-look VE. The supportive method for brainstorming consists of two parts: an idea checklist and a specification review process. In the brainstorming session, engineers use the idea checklist to review a current product and come up with individual cost-cutting ideas. By listing each item, they ensure that none are overlooked in generating ideas. If some ideas need to be further investigated, they systematically review the specifications along with the idea checklist.

The proposed method was applied to a consumer headset to demonstrate how it forms idea stimuli and reminds engineers of possible ways to approach a problem or shape a solution. A preliminary evaluation indicated that the specification review method was effective in generating ideas for later analysis. Future development of this “ideating via checklist” method is planned, beginning by generating checklists for other products. This study can be used to help those who are looking for ways to produce new and better cost-cutting ideas.

## Figures and Tables

**Table 1:** Material checklist and representative cases.

Category	Subcategory	Representative cases
Material	Same material of a lower-grade replacement	*Idea stimulus question*: Can the material be replaced with the same material of a lower grade?
		Handset upper cover_HIPS V0 resign (to HIPS HB)
		Shaft_SUS303 (to SUS204CU)
		Spacer_FOAMLEX (to FOAM PS)
		Scan frame_ABS 20% Glass Fiber (to ABS)
		Home bar switch_ABS (to ABS NTR)
		Home bar switch_ABS SCRAP (to ABS Natural)
		Cover case_ABS V0 (to ABS HB)
		Weight balance_rubber and steel (to rubber)
		User manual_wood free paper (to recycle paper)
		Locker_PPS OFL4036 (to PPS BR42B)
		Plate_SUM24L (to SUM22)
		Cover_ABS VE 0860P (to ABS VE0812)
	Different material at a lower grade replacement	*Idea stimulus question*: Can the material be replaced with a different material at a lower cost?
		Cover_ABS plastic (to HIPS)
		Pipe_Cu (to Al)
		Rubber Cover_Silicon (to SEBS)
		Pipe_Al (to Thermal Conductivity ABS)
		Motor Coil_Cu (to Al)
		Suction pipe_Cu (to Fe)
		Bimetal_SUS (to Epoxy)
		PCB pad surface plating_Ag (to Ni)
		Manual packaging vinyl (to nitron)
		Knob Damper_ABS (to HIPS)
		Guide Ice_ABS (to PP)
		Cove Case Junction_ABS V0 (to HIPS V0)
		Space EVAP_FOAM LEX (to FOAM PS)
		Tray Egg_GPPS (to PP)
		Tray Ice Cube_GPPS (to Transparency PP)
		Tray Ice Cube_HDPE (to PP)
		Tray Ice Cube_HIPS (to PP)
		Filter Tube_HIPS (to TALC PP)
		Grommet-Comp_NBR (to NR)
		Grommet-Screw_ny66 (to ABS)

**Table 2:** Form/structure checklist and representative cases.

Category	Subcategory	Representative cases
Form/structure	Simplification	*Idea stimulus question*: Can a part of the form or structure be simplified?
		Complex gasket (to flat one)
		Friction holder rear_comb pattern ribs (to simple pattern ribs)
		Belt carrier_whole sawtooth (to partial sawtooth)
		Speech circuit design_primary, speech IC and two relay (to secondary, OP Amp and one relay)
	Elimination	*Idea stimulus question*: Can a part of the form/structure be eliminated?
		Cover duct_PE foam sheet
		Fan motor transistor_Heat sink
		Paper feeder_Center area
		Wire harness of speaker assembly_EMI core
		Back up battery for power failure_lithium battery
		Main-NCU_cable tie
		Operating and Main PBA_shrink tube
	Combination	*Idea stimulus question*: Can a part of the form or structure be combined?
		Fan and box (to one piece)
		Cover_side part and top part (to one piece)
		Guide and auto document feeder (to one piece)
		Facsimile machine_rear and top cover (to one piece)
		Operating voltage_5.0 and 3.3 volts (to 3.3 volts)
	Rearrangement	*Idea stimulus question*: Can a part of the form or structure be rearranged?
		The main PBA_primary component (to optional PBA)
		PCB bottom_wire harness (to the side of the frame using pattern)
		TRIAC_bottom heat sink (to the path of the air ventilation)
		Handset Curl cord_Modular jack (to handset PBA)
	Unused Elimination	*Idea stimulus question*: Can an unused part of the form or structure be eliminated?
		Relay_unused contacts
		Hinge low guide_unused pin
		Variable resistor_unused range

**Table 3:** Graphical illustration of form/structure cases.

Subcategory	Representative cases	Before	After	Description
Simplification	Belt carrier_whole saw-tooth (to partial saw-tooth)	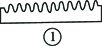	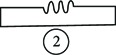	① Whole saw-tooth ② Partial saw-tooth

Elimination	Wire harness of speaker assembly_EMI core	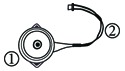	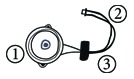	① Speaker ② Wire harness ③ EMI Core
	Power supply_heat sink	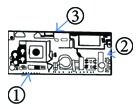	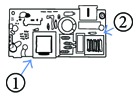	① Power supply PCB ② Frame ground circuit ③ Heat sink

Combination	Guide and Auto Sheet Feeder (to one piece)	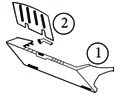	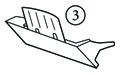	① Auto sheet feeder ② Guide ③ One piece ASF
	Cover Dust and Cover Attachment(to one piece)	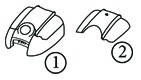	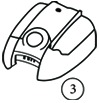	① Cover dust ② Cover attachment ③ One piece cover dust

Rearrangement	Handset Curl cord_Modular jack (to handset PBA)	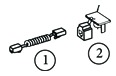	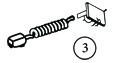	① Curl cord ② Modular jack ③ Curl cord and handset PBA are connected

**Table 4:** Dimension checklist and representative cases.

Category	Subcategory	Representative cases
Dimension	Thickness reduction	*Idea stimulus question*: Can the thickness be reduced?
		Front cover_2.0 t (to 1.5 t)
		Aluminum tape_0.3 t (to 0.2 t)
		Inlay panel_0.25 t (to 0.17 t)
		Modular jack plating_15 uin (to 13 uin)
	Length/width/height/diameter reduction	*Idea stimulus question*: Can the length/width/height/diameter be reduced?
		Wire harness length_127 mm (to 100 mm standard length)
		Antistatic brush_wires (to electrification brush only)
		Power cord length_2.5 m (to 1.8 m)
		Pipe diameter_150 mm (to 120 mm)
		PCB test point size_ø1.2 (to ø0.8)
		Spring-shaft diameter_ø1.0 (to ø0.7)
		Box height_300 mm (to 360 mm)
		PCB pattern width_12 mm (to 8 mm)
		Spring-shaft's diameter_ø1.0 (to ø0.8)
		Door harness length_260 mm (to 250 mm)
	Area/volume reduction	*Idea stimulus question*: Can the area/volume be reduced?
		User manual_183 × 295 mm (to 150 × 210 mm)
		Power supply switch_50 × 35 mm (to 18 × 20 mm)
		Main PCB_215 × 160 mm (to 180 × 120 mm)
		Power supply heat sink and frame ground circuit_55 × 150 mm (to 52 × 112 mm)
	Weight reduction	*Idea stimulus question*: Can the weight be reduced?
		Door rear cover_1.3 kg (to 1.0 kg)
		Door assembly_350 g (to 245 g)
		Packing top cushion form_800 g (to 650 g)
		Gasket door middle part_320 g (to 250 g)

**Table 5:** Component checklist and representative cases.

Category	Subcategory	Representative cases
Component	Same component of a lower grade replacement	*Idea stimulus question*: Can a component be replaced with the same component of a lower grade?
		Cable type_AWG #24 (to #26)
		SMD Chip Resister_2012 type (to 1608 type)
	Different component at a lower cost replacement	*Idea stimulus question*: Can a component be replaced with a different component at a lower cost?
		CIS Wire cable_harness type (to FFC type)
		Regulator Capacitor_tantal type (to electrolytic type)
		Bypass Capacitor_tantal type (to ceramic type)
		Battery back_backup battery (to electronic capacitor)
	Number reduction	*Idea stimulus question*: If the number of the component can be reduced
		Document TRAY_3 slots (to 3 slots)
		Exit roller_3rubbers (to 3 rubbers)
		Base frame screw_4 pieces (to 3 pieces)
		EMI bead at USB port_4 pieces (to 2 pieces)
		Zenner diode_6 pieces (to 2 pieces)
		Tank water cable tie_3 pieces (to 2 pieces)
		Bucket locking screw_4 pieces (to 2 pieces)
		Line LCD_16 × 2 line (to 16 × 1 line)
		EMI bead at flexible flat cable_2 pieces (to 1 piece)
		User's guide pages_298 pages (to 170 pages)
		Drive train gear_9 pieces (to 7 pieces)
		Power cord insert pin_3 pins (to 2 pin)
	Capacity reduction	*Idea stimulus question*: If the capacity of the component can be reduced
		Regulator_2.5 volts (to 1.8 Volts transistor type)
		Operating voltage_5.0 volts (to 3.3 volts)
		Back up capacitor_1 F (to 0.22 F)
		Program memory_8 M Byte (to 4 M Byte)
		Automatic sheet paper volume_150 sheets (to 100 sheets)

**Table 6:** Printing/coloring checklist and representative cases.

Category	Subcategory	Representative cases
Printing/coloring	Lower cost printing replacement	*Idea stimulus question*: Can a low-cost method be used to print texts?
		Internal case_labeling (to carved sealing)
		Packing box_labeling (to stamping)
		Case metalizing_solid type (to liquid type)
		Stand frame_Anodizing (to painting)
		Case_plating (to painting)
		Rail_etching (to painting)
	Coloring replacement	*Idea stimulus question*: Can a color be replaced with a natural or the same (existing) color?
		Inner surface_two different colors (to same color)
		Handle_cool white color (to natural color)
		Packaging box_four different colors (to black and white color)

**Table 7:** Processing checklist and representative cases.

Category	Subcategory	Representative cases
Processing	Joint type replacement	*Idea stimulus question*: Can the joint type be changed?
		Main PCB and body frame joint_screws (to tapes)
		Main chassis and rear cover_screws (to insert)
		Dummy ASF and Guide plate_bonding (to insert)
	Assembly step reduction	*Idea stimulus question*: Can the number of assembly steps be reduced by eliminating a component?
		Main and operating PBA connector_wire harness_(to direct soldering)
		Conduction parts_screw (to Aluminum tape)
		Chip capacitor_discrete part (to array type)
		Operating PBA and Main PBA_shrink tube (to cable tie)
	Processing step elimination	*Idea stimulus question*: Can a processing step be eliminated?
		Roller Shaft shaping_secondary operation (to primary operation)
		Case metalizing_solid type (to liquid type)
		Antifouling tape_solid film (to spray liquid)
		Inner frame_electric welding (to friction stir welding)
		PCB packaging_vacuum packaging (to normal packaging)
		Memory_OPT type (to masking type)

**Table 8:** Checklist for cost-cutting idea exploration.

Category	Subcategory	Expression
Material	Same material of a lower-grade replacement	*〈*Material, Same material of a lower-grade replacement, Shaft_SUS 303 (to HIPS HB)*〉*
	Different material at a lower grade replacement	*〈*Material, Different material at a lower grade replacement, Shaft_ABS plastic (to HIP)*〉*

Form/structure	Simplification	*〈*Form/structure, Simplification, Friction holder rear_comb pattern ribs (to simple pattern ribs)*〉*
	Elimination	*〈*Form/structure, Elimination, Cover duct_PE foam sheet*〉*
	Combination	*〈*Form/structure, Combination, Fan and box*〉*
	Rearrangement	*〈*Form/structure, Rearrangement, Main PBA_primary component (to optional PBA)*〉*
	Unused elimination	*〈*Form/structure, Unused Elimination, Relay_unused contacts*〉*

Dimension	Thickness reduction	*〈*Dimension, Thickness reduction, Inlay panel_0.25 t (to 0.17 t)*〉*
	Length/width/height/diameter reduction	*〈*Dimension, Length reduction, Wire harness length_127 mm (to 100 mm standard length)*〉*
	Area/volume reduction	*〈*Dimension, Volume reduction, Power supply switch_50 × 35 mm (to 18 × 20 mm)*〉*
	Weight reduction	*〈*Dimension, Weight reduction, Door assembly_350 g (to 245 g)*〉*

Component	Same component of a lower grade replacement	*〈*Component, Same component of a lower cost replacement, SMD Chip Resister_2012 cable (to 1608 cable)*〉*
	Different component at a lower cost replacement	*〈*Component, Different component at a lower cost replacement, CIS Wire cable_harness type (to FFC type)*〉*
	Number reduction	*〈*Component, Number reduction, Document Tray_3 slots (to 2 slots)*〉*
	Capacity reduction	*〈*Component, Capacity reduction, Regulator_2.5 volts (to 1.8 Volts transistor type)*〉*

Printing/coloring	Lower cost printing replacement	*〈*Printing/coloring, Lower cost printing replacement, Internal case_labeling (to carved sealing)*〉*.
	Coloring replacement	*〈*Printing/coloring, Coloring replacement, Inner surface_two different colors (to same color)*〉*

Processing	Joint type replacement	*〈*Processing, Joint type replacement, Main PCB and body frame joint_screws (to tapes)*〉*
	Assembly step reduction	*〈*Processing, Assembly step reduction, Main and operating PBA connector_wire harness_(to direct soldering)*〉*
	Processing step reduction	*〈*Processing, processing step reduction, Roller Shaft shaping_secondary operation (to primary operation)*〉*

**Table 9:** Specification review process.

Step 1: identifying		Step 2: exploring			Step 3: thinking
Specifications		Requirements	Who	Why and how	Cost-cutting ideas
Identify the current specifications in drawings or approved calculation sheets	If there are no current specifications, determine actual specifications through measurement	Identify regulations: laws, standards, environmental regulations, safety standards, service guidelines, and so forth	Search for the people who determine specifications: marketing, design, purchasing, manufacturing, quality, A/S, supplier, and so forth	Find any existing reason for specifications and find any existing measuring method for specifications	Think: idea form: *〈*Category, Subcategory, object (to object)*〉* By applying the idea, Q1: what problems do you expect? Q2: how do you overcome it? or Benchmarking other products Q: what specifications do you find?

**Table 10:** Schematic drawing of a handset & BOM.

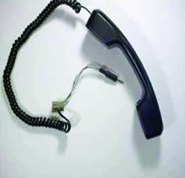			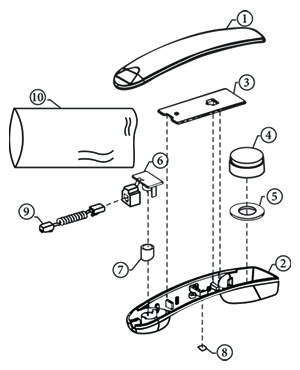

Handset			Decomposition

Bill of materials (BOM) for a handset			
Level	Parts Code	Parts name	Specifications

1	1	Handset upper	Material: ABS, color: cool white, dimensions: 164 × 44 × 25 mm, safety standard: V0

1	2	Handset lower	Material: ABS, color: cool gray, dimensions: 164 × 44 × 8 mm, safety standard: V0

1	3	Weight balance	Material: SECC-1, Weight: 2.0 Kg

1	4	Audio receiver	Electronic characteristics: 16030 OHM, 97.52 DB, dimensions: 34.8 × 17.8 mm

1	5	Ring	Material: ID17, OD35, T2, color: black, sponge

1	6	PBA SUB-etc.	PBA SUB-etc.

2	6-1	Diode-Zener (ZD1)	Part name: 1N4736A, electronic characteristics: 6.46–7.14 (5%), 1000 mW, DO-41, TP
	6-2	Capacitor film (C1)	Lead type, electronic characteristics: PEF, 4.7 nF, 5%, 100 V, TP, 5.8 × 3.1 × 12.5 mm
	6-3	MIC condenser	Electronic characteristics: 1.5 V, 0.5 mA, −44 DB, 1 kohm
	6-4	PCB handset MIC	Material: FR1, 1, 1.6, dimensions: 16.1 × 25.6 × 0.24 mm
	6-5	Handset modular jack	Material: PVC, electronic characteristics: gold plating 15 N-In, PVC AWG 26, safety standard: UL1061, housing ABS UL94V0

1	7	Rubber MIC	Material: natural, silicon rubber

1	8	Dummy handset	Material: PC, color: cool grey, dimensions: 6 × 11 × 0.25 mm, safety standard: V0

1	9	Cable form harness-handset curl cord (CFH)	Dimensions: 300 ± 20 cm, electronic characteristic: 1061 AWG26, 170

**Table 11:** Four subcategories for handset cases.

Category	Subcategory	Idea stimulus question
Material	Same material of a lower-grade replacement	Can the material be replaced with the same material, but of a lower grade?
	Different material at a lower-cost replacement	Can the material be replaced with a different material at a lower cost?

Dimension	Thickness reduction	Can the thickness be reduced?

Printing/coloring	Coloring replacement	Can a color be replaced with a natural color or another existing color?

**Table 12:** Specification review process for handset cases.

Idea generation		Specification review				
		Step 1	Step 2			Step 3
Category	Idea stimulus question	Specification	Regulation	Who	Why and how	Cost-cutting idea
Material	Can the material be replaced with the same material of a lower grade?	ABS V0	UL/TUV	Design	(i) To block a fire in the phone line (ii) Check the material's melting temperature	*〈*Material, Same material of a lower grade replacement, Headset case_ABS V0 (to ABS HB)*〉* Ans1: flammable Ans2: apply heat resistance painting
	Can the material be replaced with a different material at a lower cost?	ABS V0	UL/TUV	Design	(i) To block a fire in the phone line, (ii) Check the material's melting temperature	*〈*Material, Different material at a lower cost replacement, Headset case_ABS V0 (to HIPS)*〉* Ans1: flammable Ans2: apply heat resistance painting

Dimension	Can the thickness be reduced?	2.5 t/2.8 t	It is not broken by 5 Ns impact	Design	(i) To meet regulations (ii) Conduct an impact test	*〈*Dimension, Thickness reduction, Headset case_0.25 t*〉* Ans1: fractures before 5 Ns impact if it does not absorb impact Ans2: do not apply the idea

Printing/coloring	Can a color be replaced with a natural color or another existing color?	Special color	Aesthetics	Manufacturing	(i) To meet regulations (ii) Conduct a preference test	*〈*Printing/Coloring, Coloring replacement, Headset case_Special color (to standard color)*〉* Ans1: customers dislike colors that they recognize as standard Ans2: do not apply the idea

**Table 13:** Four subcategories for PBA SUB-etc. and CFH.

Category	Subcategory	Idea stimulus question
Form/structure	Combination	Can the handset cord be combined with the modular jack?

Dimension	Length/width/height/diameter reduction	Can the length of CFH be reduced?
	Thickness reduction	Can the gold plating in the modular handset be made thinner?

Component	Same component of a lower grade replacement	Can a component be replaced with the same component of a lower grade?

**Table 14:** Specification review process for PBA SUB-etc. and CFH.

Idea generation		Specification review				
		Step 1	Step 2			Step 3
Category	Idea stimulus question	Specification	Regulation	Who	Why and how	Cost-cutting idea
Form/structure	Can the handset cord be combined with the modular jack?	Separated handset curl cord and telephone body	Unknown	Service	(i) To be easy to repair (ii) Check the repair rate	*〈*Form/Structure, Combination, PBA SUB-etc. and CFH (to one piece)*〉* The CFH may be directly soldered onto the PCB Ans1: hard to repair Ans2: do not apply the idea

Dimension	Can the length of CFH be reduced?	300 ± 20 cm/305 cm	Unknown	Design	(i) Unknown (ii) Unknown	*〈*Dimension, Length reduction, CFH_305 cm ()*〉* Benchmarking other products Ans: similar length
	Can the gold plating in the modular handset be made thinner?	15 N-in	Maintains contact resistance after 20 insertions	Design	(i) Unknown (ii) Unknown	*〈*Dimension, Thickness reduction, Modular plating_15 N-in ()*〉* Ans1: fractures before 20 times if it is less than 15 in Ans2: do not apply the idea

Component	Can AWG26 be replaced with the same component of a lower grade?	AWG26	Unknown	Design	(i) Unknown (ii) Unknown	*〈*Component, Same component of a lower grade replacement, Wire_AWG26()*〉* Ans1: weak signal if it is less than AWG 26 Ans2: do not apply the idea

**Table 15:** Preliminary test results for the proposed method.

No.	Ideas generated during brainstorming alone	Ideas generated during brainstorming with specification review process	Improvement rate (%)
1	9	28	311
2	4	9	225
3	9	28	311
4	3	27	900
5	13	28	215
6	2	8	400
7	8	21	263
8	12	28	233
9	8	24	300
10	7	18	257

Avg.	7.5	21.9	292

## Abbreviations

ABS: Acrylonitrile butadiene styrene
ABS 20% Glass Fiber: Acrylonitrile butadiene styrene 20% glass fiber
ABS Natural: Acrylonitrile butadiene styrene Natural
ABS NTR: Acrylonitrile butadiene styrene NTR
ABS plastic: Acrylonitrile butadiene styrene plastic
ABS SCRAP: Acrylonitrile butadiene styrene SCRAP
ASF: Automatic sheet feeder
CIS: Contact image sensor
EMI: Electromagnetic interference core
FFC: Flexible flat cable
FOAM PS: Polystyrene (PS) foam
GPPS: General purpose polystyrene
HDPE: High-density polyethylene
HIPS HB: High impact polystyrene HB (horizontal burning)
HIPS V0: High impact polystyrene V0
LCD: Liquid-crystal display
NBR: Nitrile butadiene rubber
NCU: Network circuit unit
Ny 66: Nylon 66
OTP: One time programing
PBA: Printed board assembly
PCB: Printed circuit board
PE foam sheet: Polyethylene foam sheet
PP: Polypropylene
PPS: Poly phenylene sulfide
SEBS: Styrene ethylene butylenes styrene
SMD: Surface mount devices
SUM: Steel use machinability
SUS: Steel use stainless
SUS204CU: Steel use stainless 204 CU
SUS303: Steel use stainless 303
TRIAC: Triode for alternating current.
